# Editorial: Molecular pharmacological approaches against lung diseases: targeted drug discovery

**DOI:** 10.3389/fphar.2024.1419138

**Published:** 2024-07-09

**Authors:** Poonam Arora, Lalit Mohan Nainwal, Seyyed Shamsadin Athari

**Affiliations:** ^1^ SGT College of Pharmacy, SGT University, Gurugram, India; ^2^ School of Medical and Allied Sciences, G. D. Goenka University, Gurugram, India; ^3^ Department of Immunology, School of Medicine, Zanjan University of Medical Sciences, Zanjan, Iran

**Keywords:** respiratory disorders, molecular target based therapeutics, bispecific antibodies, monoclonal antibodies, RNA based therapeutics

Respiratory diseases represent a major global health and economic burden to society. The immunological complexity of asthma and chronic obstructive pulmonary disease, due to their considerable variation in phenotype and endotype, greatly hinders the identification of new therapeutic solutions (Arora et al., 2022a; Arora et al., 2022b). In recent years, increasing efforts by researchers to elucidate the molecular basis of disease pathology have coined the new concept of target-based molecular therapeutics. This approach has been exploited to tag small molecular agents obtained from biological and chemical studies that are patient-centric and have the potential to transform the course of a disease. Such therapies are efficient enough for disease treatment, mitigating side effects and reducing dose administration with better outcomes. Natural products have garnered significant attention in drug delivery due to their diverse chemical structures and inherent biological activity that can be harnessed for the development of targeted drug delivery systems (Nainwal and Arora, 2023).

The journal “Frontiers in Pharmacology” section Respiratory Pharmacology publishes high-quality research and review articles related to immunopharmacology, biology and therapeutic approaches in respiratory diseases. To introduce a special issue, the editorial “*Molecular pharmacological approaches against lung diseases: targeted drug discovery*” was published as a Research Topic in the journal, where we invited submissions ranging from original research to all types of review articles falling within the scope of the theme. The objective of this Research Topic was to shed light on the latest advancements in the use of molecular pharmacology to exploit the inherent capabilities of bioactive natural compounds for targeted drug delivery in lung diseases. A total of nine articles were selected, including original research articles, and two review articles.

The first review article by author Das et al. and collaborators explained the significant link between obesity and asthma pathophysiology. The manuscript critically discussed the therapeutic role of medicinal plants and their phytoconstituents, including celastrol, tomatidine, resveratrol, quercetin, ascorbic acid, β-carotene, chrysophanol against obesity-associated asthma. In another article, Song et al. summarized the pharmacokinetics and molecular pharmacology of a flavonoid glycoside, Baicalin, in respiratory diseases. The researchers also discussed strategies that could be utilsed to improve the bioavailability of this flavonoid. Authors, further recommend further experimental studies for development of Baicalin into pharmaceutical drug product. Blanco et al. in their manuscript reported a persistent imbalance between cell proliferation and apoptosis as an underlying factor in the pathophysiology of pulmonary arterial hypertension. The authors demonstrated the role of survivin in the pathogenesis of pulmonary arterial hypertension and the potential of YM155, a novel compound as a significant inhibitor of survivin. Phulwanti Kumari Sharma studied the potential efficacy of *Withania sominifera* extract in reducing airway inflammation in cell-based assays and experimental models of LPS-induced inflammation. The findings reported in the research paper showed reduced levels of pharmacological markers and inflammatory cytokines in the lungs of animals treated with this plant extract, suggesting a therapeutic role of the plant in inflammatory disorders.

In a paper published by Wei et al., the authors proposed the protective efficacy of a new molecule, NRICM101 against COVID-19-induced lung injury. They proposed that the therapeutic role of NRICM101 in reversing pulmonary injury may be mediated by modulating the innate immune response and inhibiting pattern recognition receptor and toll-like receptor signaling. Ye et al. validated the therapeutic efficacy of a Chinese herbal formula, ECXB, in Chronic Obstructive Pulmonary Disease. The authors utilized multiple approaches such as network pharmacology, molecular docking, and molecular dynamic simulations to identify active components in the ECXB formula. The researchers suggested that the effects of the plant were due to the multi-target synergistic actions of the plant phytocomponents.

The role of Ferroptosis has been observed in the pathogenesis of inflammation and infection. In a research paper by Wang et al., the authors discovered ferroptosis-related hub genes, CAMKK2 and CISD1 and reported them as potential immunotherapy targets and prognostic markers for asthma. Chen et al. combined a Network pharmacology-based analytical approach and *in vivo* experimental methods to explore the mechanism of Cepharanthine in the treatment of acute respiratory distress syndrome. The researchers identified novel genes that play an important part in pathogenesis of inflammatory response. A study by Jin et al. examined the effects of the inhalation of PM@Cur-RV NPs poly (lactic-co-glycolic acid) nanoparticles in the management of pulmonary diseases. For this study, they designed curcumin and resveratrol PM@Cur-RV NPs by combining poly (lactic-co-glycolic acid) nanoparticles coated with platelet membrane vesicles (PM) for targeted delivery in inflammatory lungs.

Recent advancements in understanding of the pathophysiology of chronic respiratory diseases aim towards identification of potential novel targets for pharmacological interventions. The molecular target-based therapeutics approach will open new avenues for personalised therapies that may have potential to transform patient outcomes in the management of chronic disease cases. The articles included in this Research Topic provide scientific information related to pharmacological effects of bioactives obtained from natural products that could provide endless opportunities in the design of biologically active lead molecules for utilisation in targeted drug discovery.
